# Tumor-Specific Expression of Organic Anion-Transporting Polypeptides: Transporters as Novel Targets for Cancer Therapy

**DOI:** 10.1155/2013/863539

**Published:** 2013-02-03

**Authors:** Veronika Buxhofer-Ausch, Lena Secky, Katrin Wlcek, Martin Svoboda, Valentinos Kounnis, Evangelos Briasoulis, Andreas G. Tzakos, Walter Jaeger, Theresia Thalhammer

**Affiliations:** ^1^Department of Medicine, Donauspital, Ludwig Boltzmann, Cluster for Translational Oncology, Vienna, Austria; ^2^Institute of Pathophysiology and Allergy Research, Center for Pathophysiology, Immunology and Infectiology, Medical University of Vienna, Vienna, Austria; ^3^Department of Clinical Pharmacology and Toxicology, University Hospital Zurich, Switzerland; ^4^Cancer Biobank Center, University of Ioannina, Ioannina, Greece; ^5^Department of Chemistry, University of Ioannina, Ioannina, Greece; ^6^Department of Clinical Pharmacy and Diagnostics, University of Vienna, Vienna, Austria

## Abstract

Members of the organic anion transporter family (OATP) mediate the transmembrane uptake of clinical important drugs and hormones thereby affecting drug disposition and tissue penetration. Particularly OATP subfamily 1 is known to mediate the cellular uptake of anticancer drugs (e.g., methotrexate, derivatives of taxol and camptothecin, flavopiridol, and imatinib). Tissue-specific expression was shown for OATP1B1/OATP1B3 in liver, OATP4C1 in kidney, and OATP6A1 in testis, while other OATPs, for example, OATP4A1, are expressed in multiple cells and organs. Many different tumor entities show an altered expression of OATPs. OATP1B1/OATP1B3 are downregulated in liver tumors, but highly expressed in cancers in the gastrointestinal tract, breast, prostate, and lung. Similarly, testis-specific OATP6A1 is expressed in cancers in the lung, brain, and bladder. Due to their presence in various cancer tissues and their limited expression in normal tissues, OATP1B1, OATP1B3, and OATP6A1 could be a target for tumor immunotherapy. Otherwise, high levels of ubiquitous expressed OATP4A1 are found in colorectal cancers and their metastases. Therefore, this OATP might serve as biomarkers for these tumors. Expression of OATP is regulated by nuclear receptors, inflammatory cytokines, tissue factors, and also posttranslational modifications of the proteins. Through these processes, the distribution of the transporter in the tissue will be altered, and a shift from the plasma membrane to cytoplasmic compartments is possible. It will modify OATP uptake properties and, subsequently, change intracellular concentrations of drugs, hormones, and various other OATP substrates. Therefore, screening tumors for OATP expression before therapy should lead to an OATP-targeted therapy with higher efficacy and decreased side effects.

## 1. Introduction

**Figure 1:**
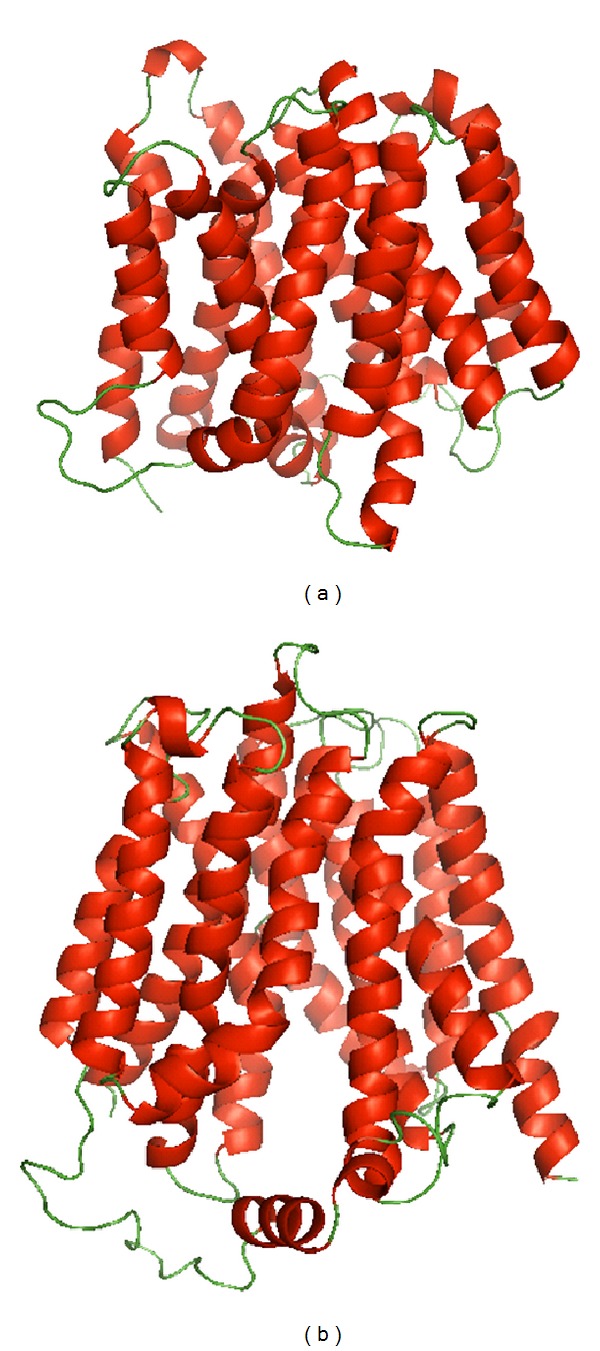
Ribbon representation of the three-dimensional model in (a) of OATP2B1 (built with modeller 9.11 using the structure template of the multidrug transporter EmrD from 2 *Escherichia coli*, pdbid: 2gfp) and in (b) of OATP1B3 (built with modeller 9.11 using the structure template of the *Escherichia coli* glycerol-3-phosphate transporter (PDB 1pw4)). The models were built by Modeller 9.11 program (San Francisco, CA, USA).

Organic anion-transporting polypeptides (OATPs) encoded by the *SCLO* genes form the SLC family 21 (OATP family) mediating the transmembrane transport of a great variety of substrates [1]. OATPs are sodium-independent plasma membrane transporters for substrates from the endogenous metabolism, such as bile acids, steroid hormone conjugates, thyroid hormones, prostaglandins, cyclic nucleotides, drugs, and xenobiotics. In humans, eleven members of the OATP family, divided into six families which share >40% amino acid sequence identity, have been identified. OATPs share a largely common structure with 12 putative transmembrane regions and a large extracellular loop between the 9th and 10th transmembrane domains (Figure 1). While the families OATP3, 5, and 6 contain only a single member, other families are further subdivided into subfamilies, which share a >60% amino acid sequence identity [2]. Members of the OATP family are expressed in a distinct pattern in excretory tissues (intestine, liver, and kidney) and on biological barriers of many organs including brain, breast, placenta, retina, ovary, and testis, where they may contribute to the absorption, distribution, and excretion of metabolic products, hormones, and drugs. OATPs work in concert with cellular metabolizing enzymes of phase 1 (cytochrome P450 isoenzymes) and phase 2 (glucuronosyltransferases, sulfotransferases, glutathione transferases, and others) enzymes as well as with efflux transporters (P-glycoprotein and breast cancer resistance protein ABCG2). The interplay between uptake, biotransformation, and efflux will strongly affect the distribution of drugs as OATP substrates [3].

There has been increasing evidence that OATPs may play an important role in the biology of various cancers. *De novo* expression of OATPs, like OATP1B1 and OATP1B3, normally only expressed in liver, has been identified in a variety of cancers (breast, colon, pancreas, stomach, prostate, bone, and ovary cancer) [4–6]. In patients with colon cancer, OATP1B3 confers resistance to anticancer drugs like paclitaxel (see Figure 3) [7]. In prostate cancer patients on androgen ablation therapy, variants of OATP1B3 with impaired function are associated with a longer progression-free and a longer overall survival, which is likely to be due to a reduced testosterone uptake into tumor cells [8, 9]. These findings recommend that therapeutic inhibition of OATP1B3 could be suitable for endocrine anticancer therapy. However, inhibiting this OATP therapeutically may interfere with normal physiological processes in the liver and impair the excretion of bilirubin, bile acids, drugs, and toxins. It may also cause drug interactions because of the inhibition of the hepatic uptake of OATP1B3 substrates and subsequently, with their biotransformation and excretion [10].

This paper focuses on the expression of OATP as a transporter for anticancer drugs and hormones in cancer. We provide an overview on the expression of specific OATPs and discuss their potential role as novel targets for anticancer therapy.

## 2. The OATP Family of Transporters

The best characterized family is the OATP1 family with three transporters OATP1A2, OATP1B1, and OATP1B3 that transport a number of typical OATP substrates including steroid hormone conjugates, thyroid hormones, prostaglandins, bile acids, and various drugs, for example, statins, antibiotics, and a number of anticancer drugs (for a review see [2]). The fourth member, OATP1C1, is regarded as thyroid hormone transporter, because of its high affinity for the thyroid hormones T_3_ and T_4_ [11]. However, it also transports steroid hormone conjugates [12].

The OATP2 family comprises two members, OATP2A1 and OATP2B1. OATP2A1 was originally identified as the prostaglandin transporter (PGT). It is thought to regulate prostaglandin (PG) levels in target tissues, for example, kidney, colon [13]. OATP2B1 has broader substrate specificity at an acidic pH (pH 6.8) for various endogenous products and drugs, while at pH 7.4, it transports mainly steroid hormone conjugates [2]. 

Typical OATP substrates (prostaglandins, thyroid hormones) are also transported by OATP3A1 and OATP4A1, but with varying affinity. For OATP3A1, transporting prostaglandins, thyroxin, vasopressin, deltorphin, and benzylpenicillin, two splice variants OATP3A1v1 and OATP3A1v2 were identified [2]. Additional substrates for the second member of the family 4, the “kidney-specific transporter” OATP4C1, which is important for the removal of uremic toxins, are cyclic nucleotides, the anticancer drug methotrexate, and other common OATP substrates, including thyroid hormones [14]. 

Transporters of the OATP family OATP5A1 and OATP6A1 are not characterized for their transport function yet. There is some evidence that OATP5A1 is involved in the chemoresistance to the oral anticancer drug satraplatin [15].

## 3. Relevance of OATP Expression in Cancer

### 3.1. The Specific Expression Pattern of OATPs in Cancer May Allow Therapeutic Targeting

**Figure 2:**
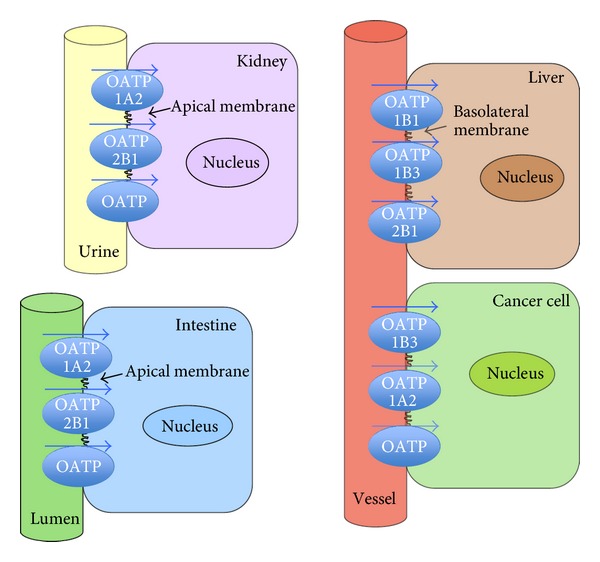
Expression of well-characterized OATPs of family 1 (OATP1A2, OATP1B1, and OATP1B3) and OATP2B1, in normal tissue and cancer cells (well-characterized OATPs are shown, and additional members of the OATP family are depicted as “OATP”).

Under physiological conditions, expression of OATP1B1, OATP1B3, and OATP6A1 is restricted to a certain tissue (Figure 2), but this pattern is no longer maintained under pathological conditions (inflammation, cancer). While in normal tissues, OATP1B1/OATP1B3 are expressed in liver and OATP6A1 in testis, the situation in cancer is different. These three OATPs are detectable in a number of different cancers. For example, “liver-specific” OATP1B3 becomes expressed in colon [16], pancreas [17], breast [18], prostate [19], lung [20], and ovarian cancer [3, 5]. “Testis-specific” OATP6A1 is highly expressed in lung [21] and brain cancer [22]. This altered expression pattern may be of a diagnostic value. It may also allow a targeted delivery of drugs. However, it has to be considered that it may also cause systemic adverse drug effects. But applying, for example, OATP1B3 substrates locally for tumors in the gastrointestinal tract or prostate, may allow an effective therapy with less side effects from the hepatic OATP1B3. Furthermore, OATP6A1-directed antibodies could be useful in the local therapy of cancers in lung, brain, and other organs expressing this OATP.

### 3.2. OATP Expression and Its Relevance for Cancer Progression

#### 3.2.1. OATPs May Affect the Intracellular Concentration of Cancer Chemotherapeutics

**Figure 3:**
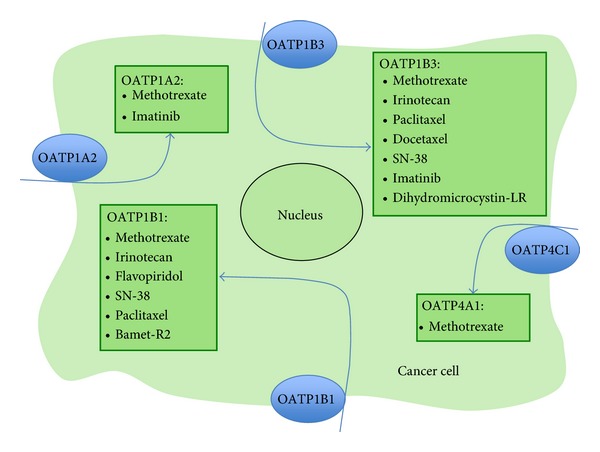
Selected anticancer drugs as substrates for organic anion-transporting polypeptides [2, 5, 6, 24].

Uptake of anticancer drugs by specific carriers plays an important role in tissue distribution, urinary and biliary excretion of drugs in healthy tissues [23]. They also provide intracellular drug concentrations necessary to reach a cytotoxic effect in cancer cells, because many cytotoxic drugs (methotrexate, taxol derivatives, imatinib, irinotecan, and flavopiridol) are substrates for OATPs (see Figure 3). 

So far, mostly OATP1A2, OATP1B1, and OATP1B3 have been carefully studied for the transport properties of anticancer drugs using *Xenopus laevis *oocytes or cancer cell lines expressing these carriers (see [6]). From the data obtained, it is obvious that a cancer-specific expression pattern of individual OATPs will influence the intracellular accumulation of drugs that are substrates for specific OATPs. Therefore, the expression pattern will influence the sensitivity of cancer cells for a certain drug. 

#### 3.2.2. OATP Confers Resistance to Apoptosis after Anticancer Chemotherapy

After camptothecin and oxaliplatin treatment, OATP1B3 overexpression provides a survival advantage for wild-type p53 expressing colon cancer cell lines by altering p53-dependent survival pathways [7].

#### 3.2.3. OATPs May Provide Steroid Hormones to Hormone-Sensitive Cancers

**Figure 4:**
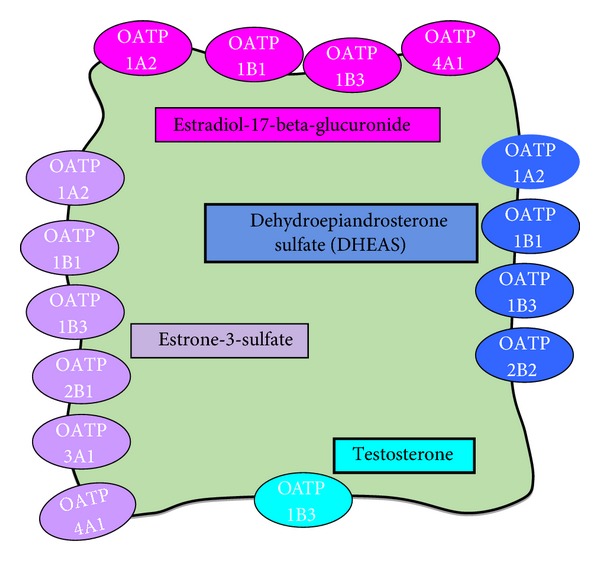
Transport of steroid hormones by OATP substrates [6].

The steroid hormone precursors, estrone sulfate (E1S), dehydroepiandrosterone sulfate (DHEAS), and the androgen testosterone, are substrates for a number of different OATPs (see Figure 4). Overexpression of these OATPs in cancer may increase the cellular levels of hormones, for example, estrogens and androgens, which drive the proliferation of hormone-dependent cancer cells. 

E1S is one of the most abundant estrogen precursors in postmenopausal women and important for the growth of estrogen-dependent breast cancer cells [25]. Seven out of eleven OATPs were found to transport E1S. For example, OATP1B3 expressed in the estrogen-dependent human breast cancer cell line MCF-7 contributes to E1S uptake [18]. The expression of steroid hormone-transporting OATP1A2, OATP1B1, OATP1B3, OATP2B1, and OATP3A1 was found to be higher in breast cancer cell lines than in the nonmalignant breast cell line MCF10A. Furthermore, specific OATP-mediated E1S uptake was observed only in malignant cells [26]. Enhanced expression of estrogen sulfates transporting OATPs may lead to the increased accumulation of steroid hormones in estrogen-sensitive tumor cells. 

OATP1A2 is also important in prostate cancer. Growth of the androgen-sensitive prostate cancer cell line LnCAP is stimulated by the androgen precursor DHEAS. The steroid hormone precursor is taken up into the cells by OATP1A2, and there, it is converted by the steroid sulfatase (STS) to active, growth stimulating DHEA. Thus, OATP1A2 together with STS is postulated to be a pharmacological target for prostate cancer treatment [27]. Other OATPs important for the growth of prostate cancer are OATP1B3, mediating the uptake of testosterone (see Figure 4), and OATP2B1, for which DHEAS is a substrate [6].

### 3.3. OATP Expression Is a Predictive Factor for the Clinical Outcome of Tumors

In some tumors, OATPs show a specific expression pattern, and there is also evidence that specific OATPs might be predictive for tumor progression. For example, OATP1B3 immunoreactivity was found to be a potent prognostic factor in human breast, prostate, and colon cancer.

## 4. OATP Expression in Breast Cancer

In breast cancer, OATP1B3 immunoreactivity was detectable in 50% of breast cancer patients. Its expression was significantly associated with a hormone-dependent growth mechanism of the breast cancer, but patients expressing this OATP had a better prognosis [28]. Also in another study, a better prognosis was seen for estrogen receptor-positive patients who expressed OATP1B3. For another E1S transporting OATP, namely OATP2B1, no relation to the clinical progression of breast cancer was found [29]. Although expression of OATPs for the transport of estrogen precursors, including E1S, would rather lead to an increased proliferation of hormone-dependent tumors, but as this OATP also transports anticancer drugs, these patients may better respond to anticancer therapy. 

Furthermore, a number of other OATPs known to transport estrogens, for example, OATP2B1, OATP3A1, and OATP4A1 were found to be expressed in breast tissue and some are reduced in malignant tissues [30]. For example, OATP3A1 was recently found to be highly important for the transport of E1S in breast cancer cell lines [26], and this may also be the case in the cancer tissue. 

It has also to be considered that apart from their role in estrogen homeostasis, expression of specific OATPs for which anticancer drugs are substrates (e.g., OATP1B1/OATP1B3 for paclitaxel) [31] may allow cancer patients to respond better to tumor therapy [28]. 

## 5. OATP Expression in Prostate Cancer

**Figure 5:**
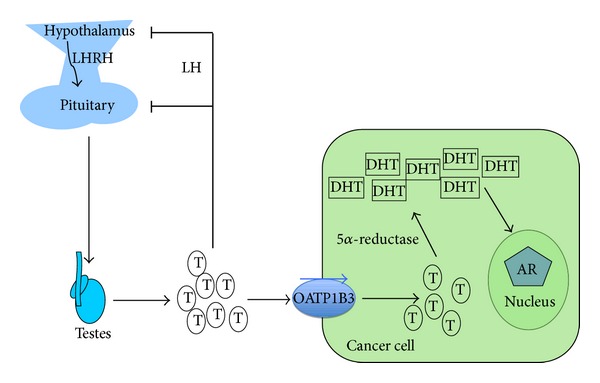
OATP1B3 provides androgens for prostate cancer cell proliferation. Production of Testosterone (T) in testis is regulated by the hypothalamic-pituitary axis via section of LHR (hypothalamic luteinizing hormone-releasing factor) and LH (gonadotropic luteinizing hormone). These hormones regulate gonadal function, including gametogenesis and synthesis of steroid hormones including testosterone (T), which is released into the circulation. It is taken up by prostate cancer cells via OATP1B3. In prostate cancer cells, T is converted into dihydrotestosterone (DHT) by 5-alpha-reductase. DHT activates the androgen receptor (AR) leading to a stimulation of cancer cell proliferation [9, 32].

Testosterone (T) deprivation therapy is important to treat advanced, androgen-sensitive prostate cancer, but it is highly variable in its effectiveness. Also acquired resistance to androgen ablation is still a major therapeutic problem. Production of testosterone in testis is regulated by the hypothalamic-pituitary axis. Secretion of hypothalamic luteinizing hormone-releasing factor in the hypothalamus and gonadotropic luteinizing hormone in the pituitary regulate gametogenesis and synthesis of steroid hormones including T in testis. T is taken up by prostate cancer cells via OATP1B3. In prostate cancer cells, T is converted into dihydrotestosterone (DHT) by 5-alpha-reductase. Activation of the androgen receptor by DHT leads to a stimulation of cancer cell proliferation (see Figure 5). Mutations in T-transporting OATP1B3 were first found to limit the response to androgen-deprivation therapy in patients [9]. 

Later, it was shown that mutations in the gene coding for OATP2B1 were also associated with time to progression. Expression of the OATP2B1 genotype, which allows a more efficient uptake of androgens into cell, is associated with enhanced tumor progression. Patients carrying mutations in OATP2B1 and OATP1B3, which allows them to import androgens more efficiently into the cancer cells, were found to have a shorter period for progression-free survival [32]. Furthermore, increased intratumoral androgen levels and an increased expression of OATP1B1, OATP1B3, OATP2A1, OATP2B1, OATP3A1 und OATP4A1 in hormone-resistant metastases compared to untreated prostate cancers was also shown [9].

In line with these findings, the risk for androgen ablation-insensitive metastases is increased in patients with variant alleles for OATP2B1 or OATP1B3. The data so far suggest that OATPs could be potential biomarkers for assessing risk of androgen-insensitive metastases in patients who should be treated earlier with a non-hormonal based anticancer therapy [9].

## 6. OATP Expression in Colorectal Cancer

Using tissue microarrays, OATP1B3 immunoreactivity was detectable in the majority (56%) of colon tumor samples from 278 patients with all tumor stages. Similar to prostate cancer where expression OATP1B3 is significantly related to the Gleason score as a marker for tissue dedifferentiation [5], higher OATP1B3 levels in colon are associated with earlier tumor stage and they are found in better differentiated tumors. However, they are not predictive for the 5-year survival and for tumor recurrence. Within lower tumor grades, OATP1B3 expression is associated with an improved 5-year survival, while the tumor recurrence in patients with poorly differentiated tumors is independent on the expression of this OATP [16].

## 7. OATP Expression in Pancreatic Cancer

Extensive research has failed to produce any therapy efficient enough to substantially extend the median survival of treated patients beyond 6 months. Currently available therapies remain palliative on their intent [33–35]. Therefore, identification of new molecular targets and discovery of novel targeted therapies is of top priority for pancreatic cancer research. In a recent study, the expression of OATP1A2, OATP1B1, and OATP1B3 was studied by immunohistochemistry in a sample of 12 patients as well as on the mRNA level in two pancreatic cancer cell lines [17]. Quantitative analysis was done by the HistoQUEST Software using the TissueFaxs Microscopic Image Analysis System (TissueGnostics, Vienna, Austria). The three studied polypeptides were found ubiquitously expressed in all studied pancreatic cancer biopsy samples. Methods used confirmed extensive immunostaining of the entire cancer cell tissue with the antibodies against these OATPs. In detail, the OATP1A2 expression signal was weak in one sample and moderate to strong in all others. OATP1B1 was found to be weakly expressed in all 12 cases. Immunostaining with the mMDQ antibody against OATP 1B1/1B3 was proved to be the most intense. Nine cases demonstrated moderate expression and three cases stained strong. OATP 1B1 and 1B3 mRNA expression in two cell lines, MIA PaCa-2 and Bx-PC3, was comparable to that in normal liver, which was taken as a control, because both of these transporters are considered “liver-specific”. Their mRNA expression, however, in normal pancreas was either undetectable (OATP 1B1) or 30–60 times lower than that in normal liver (OATP 1B3). 

The OATPs investigated in this study were all found to be ubiquitously expressed at the protein and the mRNA level which flags them as appropriate candidates for *in vitro* studying of OATP-targeted anticancer compounds [17].

## 8. OATP Expression in Liver Cancer

In tumors of the liver, the expression of OATP1B1 and OATP1B3 is reduced along the degree of tissue dedifferentiation. This could reflect the reduction of metabolic function of liver cells in more advanced tumors [5]. In hepatocellular carcinoma patients, which undergo liver transplantation, expression levels of these OATPs are negatively related to tumor-related death after recurrence, but the expression of the OATPs is not correlated to the regression-free survival [36]. On the other hand, we showed that some OATPs (OATP2A1, OATP3A1, OATP4A1, and OATP5A1) become upregulated in primary and metastatic liver cancer as compared to nonmalignant liver. In these patients, OATP-derived immunoreactivity is located in the plasma membranes and, occasionally, in the cytoplasm of tumor cells. In some tumors, staining is also seen in bile duct cells and in stromal cells [37]. This pattern suggests that particular OATPs might be necessary to supply tumor cells with nutrients, hormones, or tissue factors in cells working in a close interaction between the tumor and its environment. These OATPs might be further exploited for the discovery of novel anticancer agents [38]. 

## 9. Members of the OATP Family: Role in the Transport of Anticancer Drugs and Hormones 

### 9.1. OATP1A2

OATP1A2 (gene symbol, SLCO1A2) mediates the cellular uptake of a wide range of endogenous substrates including estrogen conjugates, DHEAS, thyroid hormones, prostaglandins, and bile acids. These groups are “typical OATP substrates” as they are transported by the vast majority of OATPs. But there is a diverging affinity for individual drugs. Figure 3 gives an overview on anticancer drugs as substrates for individual OATPs (see [2]).

OATP1A2 is also a transporter for many drugs, including statins, morphine derivates, and antibiotics. Importantly, the folate antimetabolite methotrexate and imatinib, a drug applied for many forms of leukemia, are OATP1A2 substrates [6]. 

This OATP is highly expressed at physiological barriers, for example, blood-brain barrier, the brush border membrane of the distal nephron, bile duct cells, and endothelial cells of the blood-brain barrier, and in the apical membranes of epithelial cells in the small intestine, suggesting a particular role of this OATP in drug disposition. OATP1A2 levels are low in all regions of the intestine [38, 39], but the transporter colocalizes with MDR1 to the brush border domain of enterocytes [40]. Therefore, OATP1A2 could be of pharmacological relevance if the levels of this OATP are induced by pharmacological administration of, for example, Vitamin D(3) or pregnane-X-receptor (PXR) ligands [41]. The bioavailability of oral applied anticancer drugs, for example, imatinib, and drugs given frequently to cancer patients, like deltorphin II and nadolol, could be influenced by the induction of intestinal OATP1A2 [42]. Interaction between OATP1A2 substrates may change intracellular concentrations of drugs which may influence the efficacy of the therapy and/or lead to serious side effects.

According to its localization in the basolateral membrane of the distal nephron, it may also regulate the renal excretion of anticancer drugs. This is suggested from the finding that SLCO1A2 mutations influence the imatinib clearance in patients with chronic myeloid leukemia [42]. On the other hand, another study reported that the imatinib absorption was not related to OATP1A2 variants [43]. Also methotrexate is mostly excreted via the kidney, and OATP1A2 may mediate the tubular reabsorption of methotrexate. Altered expression of OATP1A2 in the kidney may therefore contribute to drug-induced toxicity. Whether OATP1A2 mutations may influence drug clearance in patients is not known yet but could occur, as methotrexate transport is altered in *Xenopus laevis* oocytes expressing different OATP1A2 variants [44].

Finally, high expression levels of OATP1A2 in tumor cells in breast, prostate, and bone cancer will influence cellular levels of anticancer drugs imatinib and methotrexate and determine their local efficacy [26, 27].

The ligand-activated transcription factor PXR is known to play a role in the regulated expression of drug metabolizing enzymes and transporters. Data in breast cancer revealed that the PXR activator rifampicin can stimulate OATP1A2 expression. On the other hand, a statistical analysis of data from approximately 100 patients suggests that variations in genes coding for PXR, OATP1A2, and the OATPs 1B1, 1B3, and 2B1 do not contribute to breast carcinogenesis [45]. At the protein level, protein kinase C was shown to regulate the correct insertion of OATP1A2 into the plasma membrane in part by clathrin-dependent pathways. Inhibition of PKC activity blocks the transport function of this OATP [46, 47].

### 9.2. OATP1B1/1B3

OATP1B1 and OATP1B3 are highly expressed in normal liver and are regarded as “liver-specific” OATPs. OATP1B3 is also expressed in various human cancer tissues, and some studies suggest that its expression levels are associated with the prognosis and clinical outcome of tumors [28, 48].

Both transporters from the 1B family are carriers for typical OATP substrates including hormones and conjugates, bile acids, statins, antibiotics, and a number of other drugs [2]. Also some anticancer drugs, for example, methotrexate, docetaxel, the irinotecan metabolite SN-38, and the immunosuppressive drug rapamycin, are transported by both OATPs (reviewed in [6]). The campthocetin derivatives gimatecan and BNP1350 [49], the cyclin-dependent kinase inhibitor flavopiridol [50], and the cisplatin bile acid derivatives Bamet-R2 [cis-diamminechloro-cholylglycinate-platinum(II)], Bamet-UD2 [cis-diammine-bisursodeoxy-cholate-platinum(II)] [51] are substrates for OATP1B1. Substrates for OATP1B3 are the Her-2 tyrosine kinase inhibitor CP-724,714 [52], imatinib [53], and PKI166, a specific inhibitor of the tyrosine kinase activity of two epidermal growth factor receptors [54].

Both OATPs are polymorphic, and, so far, a number of variants with altered drug affinity and transport kinetics were identified and characterized *in vitro *(reviewed in [55, 56]). Expression of different variants in patients may alter the bioavailability of anticancer drugs as shown in animal studies, where absence of the analog of human OATP1B1/1B3 in mice led to a decrease in the docetaxel clearance. However, in patients, the reduced function of OATP1B1 or OATP1B3 variants did not alter docetaxel clearance. Therefore, only functional defects in both OATPs may influence the disposition of docetaxel [57]. Uptake of SN-38 was reduced in cell lines expressing three common variants of OATP1B1. An influence on the pharmacokinetics of SN-38 was also proposed for patients with the respective variants [58]. Indeed, patients with the SLCO1B1∗15 polymorphism had lower clearance of irinotecan [59].

Gadoxetic acid, which is used for liver magnetic resonance imaging in patients with liver cancer, is also an OATP1B1/OATP1B3 substrate. Although the pharmacokinetic characteristics for the drug were not influenced by SNP, in people carrying certain OATP1B1 variants, the magnetic resonance imaging signals were disturbed [60]. 

OATP1B1 and OATP1B3 expressions were shown to be reduced in primary and metastatic liver cancer. However, OATP1B3 is expressed in many cancers, for example, in colon, breast, pancreas, ovary, testis, bladder, prostate, and so forth [5], where it may influence tumor growth and survival in an organ-specific way [61]. Overexpression in colon cancer may contribute chemoresistance as it promotes the survival of colon cancer cells after treatment with anticancer drugs dependent on p53 expression [7]. In ovarian cancer cell lines, OATP1B1 and OATP1B3 were identified as high-affinity paclitaxel transporters. As both OATPs are expressed in 50% of cancer samples, they might have a role in the disposition of paclitaxel during first-line therapy of ovarian cancer [31].

Although OATP1B3 is frequently found in tumors, the molecular entity of cancer-associated OATP1B3 is still poorly addressed. Recently, a new OATP1B3 mRNA variant named cancer-type OATP1B3 was identified and found to be highly expressed in colon and lung cancer specimens. However, the translation product of this gene has not been characterized yet, and therefore, no statement on its impact on cancer growth and progression can be made [62].

By mediating the uptake of steroid hormones in hormone-sensitive tumor cells, these OATPs may promote the cell survival. OATP1B3 expression is regulated by transcription factors like the farnesoid-X-receptor (FXR), the hepatocyte nuclear factor (HNF) 1-alpha, and HNF3-beta. HNF1-alpha and HNF3-beta might contribute to its liver-specific expression, and FXR might play a role in its transcriptional activation by bile acids [63].

### 9.3. OATP1C1

OATP1C1 is a transporter with the highest affinity for thyroid hormones, and it could be important for the transport of these hormones in target tissues. Although it has some affinity for other OATP substrates, no cancer drugs were identified to be transported by this OATP. It is expressed in bone tumors too [64]. OATP1C1 might also contribute to the excretory system of the colon [65].

### 9.4. OATP2A1

The prostaglandin transporter OATP2A1 is widely expressed in different organs (e.g., brain, gastrointestinal tissues, kidney, heart, liver, ovary, lung, prostate, skeletal muscle, and spleen) [66]. At the protein level, OATP2A1 was detected in the luminal membrane of endothelial cells forming the blood-brain barrier and the blood-tumor barrier [67], in the pyloric glands of the antrum and in parietal cells in the gastrointestinal tract [68], as well as in the luminal and glandular epithelium of the endometrium [69]. The prostaglandin carrier mediates the transport of several prostanoids including prostaglandin E(2) and PGF(2-alpha).

High mRNA expression was detected in many other tumors including cancers of breast, liver, ovary, lung, and bone. It was shown to be downregulated at the mRNA and protein level in colorectal cancer, where it seems to contribute to the regulation of extracellular proinflammatory PGE(2) levels [70]. PGE(2) is taken up into cells from the extracellular milieu by OATP2A1, where it can be inactivated by oxidation to inactive 15-keto PGE(2) by the 15-hydroxyprostaglandin dehydrogenase [66].

### 9.5. OATP2B1

The ubiquitously expressed OATP2B1 has a high affinity for steroid hormone conjugates; OATP2B1 transports other OATP substrates including thyroid hormones, PGE(2), and many drugs. No anticancer agents were identified as a substrate for OATP so far. OATP2B1 expression was found to be regulated by steroid hormones. Progesterone was shown to stimulate OATP2B1-mediated transport of precursors for steroid hormone synthesis, E1S, DHEA, and pregnenolone sulfate, but not of other OATP substrates [71]. 

OATP2B1 expression was also demonstrated in human gliomas, where it was localized to endothelial cells at the blood-brain barrier and blood-tumor barrier [72]. Increased expression was found in breast cancer specimens as compared to nonmalignant breast [30]. In breast cancer, its expression increases with increased tumor grade [29]. Furthermore, OATP2B1 mRNA expression was higher in bone cysts than in osteosarcoma tissues [64].

### 9.6. OATP3A1

OATP3A1 was shown to transport hormone and conjugates, prostaglandins, vasopressin, and benzylpenicillin and other antibiotics. Highest levels of this OATP were found in testis, brain, lung, spleen, human osteoblast-like cells, and bone-marrow stromal cell. High levels of this OATP were found in breast cancer, where it was detected in the membrane and cytoplasm of malignant cells in breast tumor specimens [73].

### 9.7. OATP4A1

The expression pattern of OATP4A1 is similar to that of OATP3A1. OATP4A1 is highly expressed in various carcinomas, for example, breast, lung, colon, and ovarian carcinoma, and metastatic tumors of colorectal cancer in liver. OATP4A1 and also OATP2B1 are significantly highly expressed in the colon of patients with inflammatory bowel disease than in normal colonic tissue [38]. In colorectal neoplasia, increased expression of prostaglandin E(2) transporting OATP4A1 and OATP2B1 may lead to a decreased sensitivity to cyclic nucleotides [65].

### 9.8. OATP4C1

OAT4C1 is expressed mainly in the kidney but is also found in some tumors, for example, colon. Human OATP4C1 were previously shown to transport cardiac glycosides, thyroid hormone, cAMP, and methotrexate [24]. In the rat kidney, Oatp4c1 reduced hypertension, cardiomegaly, and inflammation in the setting of renal failure. This was related to its excretory function in kidney. SLCO4C1 overexpression decreased plasma levels of the uremic toxins, for example, guanidino succinate, and dimethylarginine [74]. Statins, which act as inducers of nuclear aryl hydrocarbon receptors, upregulate SLCO4C1 transcription [75].

### 9.9. SLCO5A1

This poorly characterized OATP was detected at the mRNA levels in many tissues including heart, skeletal muscle, brain, breast, and blood cells. At the mRNA level it was described in cancers of the liver, bone, and breast. In normal breast tissue, OATP5A1 is located at the cell membrane of epithelial cells lining the milk ducts. In breast cancer, OATP5A1 loses the membrane localization as immunoreactivity was also visible in the cytoplasm of milk duct cells [73]. 

Haploinsufficiency of the gene coding for OATP5A1 together with that encoding the heparan sulfate 6-O-endosulfatase 1 acting as a regulator of numerous growth factors in skeletal embryonic development were found to cause a rare autosomal dominant disorder, the mesomelia-synostoses syndrome. It is characterized by mesomelic limb shortening, acral synostoses, and multiple congenital malformations [76]. 

### 9.10. OATP6A1

OATP6A1 was originally identified as a cancer/testis (CT) antigen strongly expressed at the mRNA level only in normal testis. Weak expression was seen in spleen, brain, and placenta [22]. Like other CT antigens, OATP6A1 is expressed in a number of cancers (brain, urinary bladder, and lung). Because of its high immunogenic potential, these CT antigens would be of potential utility as a target for antibody-based therapy for a variety of tumor types [77].

## 10. Regulation of OATP Expression

Altered expression of OATPs after malignant transformation of tissues raises the question about mechanisms involved in the regulation of the expression of these transporters. Although data on regulatory mechanisms for the expression of OATPs are still rare, regulation of OATP1B1, OATP1B3, OATP2A1, OATP2B1, and OATP4A1 were studied on the transcriptional and posttranscriptional levels. Activation of transcription factors, DNA-dependent gene silencing, and posttranscriptional modifications are involved in the regulation of their expression [31]. In cancer, these processes may change the expression levels of transporters and/or shift the transporter from the plasma membrane to cytosolic compartments leading to changes in OATP functional properties [6]. 

Transcriptional regulation by different nuclear receptors plays an important role in the regulation of OATP expression. For example, in breast carcinoma tissue and cancer cell lines, expression of OATP1A2 is closely correlated to the expression of the pregnane-X-receptor (PXR) [78]. This chemosensitizing nuclear receptor is activated by a wide range of drugs and xenobiotics, including rifampicin, capable to induce OATP1A2 in these cell lines [79]. In the breast cancer cell line T47-D, induction of OATP1A2 by rifampicin was accompanied by an increased cell proliferation, for which increased uptake of E1S could be responsible. Both increased uptake and cell proliferation after rifampicin can be inhibited by the application of PXR antagonists. Also expression of OATP1B3 and OATP1B1 are regulated by nuclear receptors, but in contrast to OATP1A2, PXR activation by rifampicin inhibits OATP1B3 expression. 

Alterations in the cytokine pattern by viral infection and inflammation reduce the expression of OATP1B1, OATP1B3, and OATP2B1 as studies in patients suffering from hepatitis C showed [80]. Interferon gamma, tumor necrosis factor-alpha, interleukin-1-beta, and -6 were shown to decrease mRNA expression of the three OATPs in isolated hepatocytes [81].

DNA methylation-dependent gene silencing was demonstrated to regulate the expression of OATPs. Holla et al. showed [70] that the application of a demethylating agent or a histone deacetylase inhibitor could partially restore the reduced expression of OATP2A1 in colorectal cancer cell lines. Furthermore, the analysis of the DNA methylation profile of OATPs in colon cancer cell lines revealed that GpG nucleotides around the transcriptional start site of OATP1B3 possess different methylation patterns leading to distinct expression pattern in these cell lines [82].

On the posttranscriptional level, phosphorylation and glycosylation may alter subcellular localization of the OATPs. For example, altered phosphorylation in breast cancer shifts the OATP2B1 from the plasma membrane to cytosolic compartments. Activation of protein kinase C by phorbol ester results in increased OATP2B1 phosphorylation, subsequent rapid clathrin-dependent internalization, and lysosomal degradation [46, 47].

## 11. Conclusions

The physiological expression pattern of OATPs is altered in malignancies. As many anticancer drugs are substrates for OATPs, expression of these transporters in tumors may affect the intracellular concentration of drugs, and, therefore, influence their effectiveness. Furthermore, expression levels of these influx transporters, known to work in concert with efflux transporters and drug-metabolizing enzymes, respectively, may play a crucial role in chemoresistance mechanisms. OATPs may also influence hormone-dependent tumor growth, because they mediate the uptake of steroid hormone precursors (E1S, DHEAS) into cancer cells. These precursors can be converted to the active estrogens and androgens by steroid sulfatase and 17-beta-dehydrogenases, respectively. 

Furthermore, OATPs, highly and exclusively expressed in certain cancer types, may serve as novel biomarkers for the response to anticancer drugs and/or hormonal therapy. Whether OATPs may be targets for cancer-directed therapy has to be further evaluated. 

Further research is also required to determine these transporters in individual tumors. *In vivo *models are necessary to investigate their potential to alter the response to clinically established and novel anticancer substrates as well as to therapeutically applied hormones. Expanding our understanding of the different expression patterns of OATPs in tumors will finally aid oncologists when prescribing anticancer drugs known to be transported by OATPs. 

## Abbreviations

Bamet-R2: cis-Diamminechloro-cholylglycinate-platinum(II)
Bamet-UD2: cis-Diammine-bisursodeoxy- cholate-platinum(II)
C/T: Cancer/testis antigen
DHT: Dihydrotestosterone
HNF: Hepatocyte nuclear factor
FXR: Farnesoid-X-receptor
LH: Gonadotropic luteinizing hormone
LHR: Hypothalamic luteinizing hormone-releasing factor
OATP: Organic-anion transporting polypeptide
SLCO: Solute carrier for organic anions, OATP protein
PG: Prostaglandin
PXR: Pregnane-X-receptor
SN-38: Active metabolite of irinotecan
T: Testosterone
A: Androgen receptor (AR).
